# Coexpression Network Analysis Identifies a Novel Nine-RNA Signature to Improve Prognostic Prediction for Prostate Cancer Patients

**DOI:** 10.1155/2020/4264291

**Published:** 2020-09-01

**Authors:** Jiarong Cai, Zheng Chen, Xuelian Chen, He Huang, Xia Lin, Bin Miao

**Affiliations:** ^1^Department of Urology, The Third Affiliated Hospital of Sun Yat-sen University, Guangzhou, Guangdong 510630, China; ^2^Department of Stomatology, The Third Affiliated Hospital of Sun Yat-sen University, Guangzhou, Guangdong 510630, China; ^3^General Surgery Department, The Third Affiliated Hospital of Sun Yat-sen University, Guangzhou, Guangdong 510630, China; ^4^Department of Gynecology, The Third Affiliated Hospital of Sun Yat-sen University, Guangzhou, Guangdong 510630, China; ^5^Department of Organ Transplantation, The Third Affiliated Hospital of Sun Yat-sen University, Guangzhou, Guangdong 510630, China

## Abstract

**Background:**

Prostate cancer (PCa) is the most common malignancy and the leading cause of cancer death in men. Recent studies suggest the molecular signature was more effective than the clinical indicators for the prognostic prediction, but all of the known studies focused on a single RNA type. The present study was to develop a new prognostic signature by integrating long noncoding RNAs (lncRNAs) and messenger RNAs (mRNAs) and evaluate its prognostic performance.

**Methods:**

The RNA expression data of PCa patients were downloaded from The Cancer Genome Atlas (TCGA) or Gene Expression Omnibus database (GSE17951, GSE7076, and GSE16560). The PCa-driven modules were identified by constructing a weighted gene coexpression network, the corresponding genes of which were overlapped with differentially expressed RNAs (DERs) screened by the MetaDE package. The optimal prognostic signature was screened using the least absolute shrinkage and selection operator analysis. The prognostic performance and functions of the combined prognostic signature was then assessed.

**Results:**

Twelve PCa-driven modules were identified using TCGA dataset and validated in the GSE17951 and GSE7076 datasets, and six of them were considered to be preserved. A total of 217 genes in these 6 modules were overlapped with 699 DERs, from which a nine-gene prognostic signature was identified (including 3 lncRNAs and 6 mRNAs), and the risk score of each patient was calculated. The overall survival was significantly shortened in patients having the risk score higher than the cut-off, which was demonstrated in TCGA (*p* = 5.063E − 03) dataset and validated in the GSE16560 (*p* = 3.268E − 02) dataset. The prediction accuracy of this risk score was higher than that of clinical indicators (the Gleason score and prostate-specific antigen) or the single RNA type, with the area under the receiver operator characteristic curve of 0.945. Besides, some new therapeutic targets and mechanisms (MAGI2-AS3-SPARC/GJA1/CYSLTR1, DLG5-AS1-DEFB1, and RHPN1-AS1-CDC45/ORC) were also revealed.

**Conclusion:**

The risk score system established in this study may provide a novel reliable method to identify PCa patients at a high risk of death.

## 1. Introduction

Prostate cancer (PCa) is the most frequently diagnosed malignancy in men, with an estimated 174,650 new cases and 31,620 deaths in 2019 in the United States [1]. Although diverse treatment strategies (including radical surgery, radiotherapy, chemotherapy, and androgen deprivation therapy) have been demonstrated to be effective, approximately 25% of PCa patients will experience recurrence, metastasis, and develop into castration-resistant PCa, leading to the poor overall survival (OS) [2]. Therefore, it is critical to early identify the patients who are at a high risk for death. Serum level of prostate-specific antigen (PSA) [3], the Gleason score [4], and tumor, node, and metastasis (TNM) staging [5] are the routine clinical indicators for the prediction of OS in patients with PCa. Nevertheless, in clinic, some scholars also observed that patients with the same stage could progress to opposite consequences [6], while similar prognostic outcomes were present in patients with different PSA levels or Gleason scores [7, 8]. Therefore, identification of more effective prognostic biomarkers is highly desirable.

With the development of molecular biology, recent studies have attempted to develop the gene molecular signature for the prognostic prediction. Some have been demonstrated to possess a higher predictive power than the above clinical indicators. For example, Li et al. developed a risk score constructed by 6 protein-coding genes. Univariate and multivariate Cox regression analyses showed that this risk score was independent of TNM stage for biochemical recurrence- (BCR-) free survival prediction (hazard ratio (HR) = 3.045, 95% confidence interval (CI) = 1.655 − 5.602; *p* < 0.001). A subgroup analysis revealed that there were also significant survival differences when the patients with the same Gleason score (≤7 or >7) were classified into the high-risk score group and the low-risk score group [9]. Shi et al. established a prognostic risk score based on 9 protein-coding genes. Receiver operating characteristic (ROC) analysis indicated that the prediction accuracy of this risk score for BCR-free survival was higher than that of Gleason score (area under curve (AUC) = 0.836 vs.0.742) and pathological T stage (AUC = 0.836 vs.0.780) [10]. Similar superiority of the molecular risk score was also observed in the study of Huang et al. who found the four-long noncoding RNA- (lncRNA-) based risk score was independent of the American Joint Committee on Cancer T stage and Gleason score for the prediction of BCR-free survival and disease-free survival. The prediction accuracy for both 2- (AUC = 0.823 vs.0.787) and 5-year BCR (AUC = 0.833 vs.0.797) can be improved by 3.6% if the four-lncRNA signature was added to the clinical indicators [11]. Xu et al. identified an eight-lncRNA signature as an independent factor associated with BCR-free survival and demonstrated its prognostic ability was better than that of the Gleason score (AUC = 0.79 vs.0.688) and positive lymph node (AUC = 0.79 vs.0.622) [12]. However, all of these studies were exploring the prognostic value of a single RNA type. Previous studies on other cancers indicated the lncRNA-mRNA combined signature seemed to be more effective than the lncRNA or mRNA alone for the prognostic prediction [13, 14]. Hence, it is essential to further develop an lncRNA-mRNA integrated prognostic signature for PCa. Furthermore, most of the studies involving comparison with clinical indicators focused on the prediction for BCR-free survival, not for OS, which was also a novelty of our study.

The objectives of the current study were (1) to establish a signature for OS prediction based on the PCa-driven lncRNAs and mRNAs which were screened by weighted gene coexpression network analysis (WGCNA) [15], a systematic biological method to cluster highly correlated genes; (2) to confirm the superiority of the integrated molecular signature to clinical indicators or single molecular type; and (3) to reveal the underlying functions of the gene signature.

## 2. Materials and Methods

### 2.1. Data Collection and Preprocessing

The level-3 RNA-seq dataset of PCa patients was downloaded from The Cancer Genome Atlas (TCGA, https://portal.gdc.cancer.gov/) on October 18, 2019, including 494 tumor samples (which had survival outcomes) and 54 control samples. This dataset was selected as the training dataset for module and signature identification. The normalized fragments per kilobase of exon per million fragments mapped (FPKM) were used to represent the expression of gene. Furthermore, three gene microarray datasets were downloaded from Gene Expression Omnibus (GEO, http://www. http://ncbi.nlm.nih.gov/geo/) because they also analyzed the gene expression profiles in tumor and control tissues of PCa patients (GSE17951: control, *n* = 14; tumor, *n* = 109 [16, 17] in which some samples without clear type description were deleted; GSE70768: control, *n* = 73; tumor, *n* = 126 [18]; these two datasets were used to estimate module preservation) or recorded the prognostic outcomes in all PCa patients (GSE16560: tumor, *n* = 281 [19]; this dataset was used for signature validation). The series matrix files were collected from GEO, and the probe IDs were converted into gene symbols via corresponding platforms (GSE17951: GPL570, [HG-U133_Plus_2] Affymetrix Human Genome U133 Plus 2.0 Array; GSE70768: GPL10558, Illumina HumanHT-12 V4.0 expression BeadChip; GSE16560: GPL5474, Human 6k Transcriptionally Informative Gene Panel for DASL).

The known mRNAs and lncRNAs in the above sequencing or microarray datasets were reannotated by the HUGO Gene Nomenclature Committee (HGNC; http://www.genenames.org/) which contains the official nomenclature for 4,495 lncRNAs and 19,219 protein-coding genes [20]. RNAs with a median expression value equal to 0 were removed. Only the mRNAs and lncRNAs that were annotated in all included datasets were used for the following analyses.

### 2.2. Screening of PCa-Driven Modules

The WGCNA package in R (version 1.61; https://cran.r-project.org/web/packages/WGCNA/index.html) [15] was used to identify PCa-driven modules. Briefly, the expression and connectivity correlations of all RNAs between any two datasets (TCGA, GSE17951, and GSE7076) were first calculated to confirm their comparability. Then, the soft threshold power (*β*) was selected using the pickSoftThreshold function according to the scale-free topology criterion, with which the weighted adjacency matrix was generated and the gene dendrogram was constructed based on topological overlap matrix dissimilarity. Next, modules with a cutHeight of 0.995 and minSize ≥ 50 were identified using TCGA data via the dynamicTreeCut method [21]. The preservation of the identified modules was validated in the GSE17951 and GSE7076 datasets using the modulePreservation statistics [22]. Preservation *Z* − score > 5 implied the corresponding module was preserved. Finally, the potential function of preserved modules was analyzed according to the userListEnchment function, while their clinical association was investigated by moduleTraitCor and moduleTraitPvalue algorithms in the WGCNA package.

### 2.3. Screening of Differentially Expressed lncRNAs and mRNAs

The differentially expressed lncRNAs (DELs) and mRNAs (DEGs) between PCa and normal controls were screened using the MetaDE.ES function in the MetaDE package (version 1.0.5, https://cran.r-project.org/web/packages/MetaDE/). Briefly, due to the presence of the platform difference in three datasets (TCGA, GSE17951, and GSE7076), the heterogeneity of RNAs across them was first assessed by tau^2^ statistic and Chi-square-based Q-test. Only the RNAs with homogeneity (tau^2^ and Q *p* value > 0.05) were included for the differential analysis. The gene expression difference was determined by the MetaDE.pvalue algorithm, with the false discovery rate (FDR) < 0.05 set as the significance threshold. Furthermore, the log2FC (fold change) of RNAs in each dataset was also calculated. Only the RNAs with the consistency in significance and differential trend in three datasets were considered as differentially expressed RNAs (DERs).

### 2.4. Construction of an lnRNA-mRNA Prognostic Model

A Venn diagram (http://bioinformatics.psb.ugent.be/webtools/Venn/) was developed to identify the overlap between PCa-driven module RNAs and DERs, which were used as seeds for the following survival analysis. The association between the expression levels of DERs and OS was first evaluated by the univariate Cox proportional hazards regression analysis in the “survival” package of R (version, 2.41-1; http://bioconductor.org/packages/survivalr/). Only DERs with log-rank *p* < 0.05 were considered to have the prognostic potential, which then underwent the multivariate Cox regression analysis to assess their independence. In order to confirm whether the signature identified by multivariate analysis was optimal, L1-penalized (least absolute shrinkage and selection operator (LASSO)) Cox proportional hazard model in the penalized package (version, 0.9-5; http://bioconductor.org/packages/penalized/) [23, 24] was further applied. The risk score formula was generated based on the expression levels of prognostic RNAs (Exp_DERs_) and their LASSO coefficients (*β*_DERs_):

(1) $$\begin{array}{c} \text{Risk score}=\beta_{\text{lncRNA}1}\times {\text{Exp}}_{\text{lncRNA}1}+\beta_{\text{lncRNAn}}\times {\text{Exp}}_{\text{lncRNAn}}+\beta_{\text{mRNA}1}\times {\text{Exp}}_{\text{mRNA}1}+\cdots \beta_{\text{mRNAn}}\times \text{Ex}{\text{p}}_{\text{mRNAn}}. \end{array}$$

The risk score of each patient was calculated according to the above formula, and then, the patients were divided into the low-risk group and the high-risk group using the median score as the cut-off point. The prognostic effect of the risk score was examined by the Kaplan–Meier estimate (log-rank *p* value < 0.05) and ROC analysis (AUC = 0.5 ~ 1; the AUC towards 1 indicated a good performance). Furthermore, univariate, multivariate Cox regression, and subsequent stratification analyses were also conducted to estimate the association between the risk score and clinical pathological characteristics, with the criteria of statistical significance set as *p* < 0.05.

### 2.5. Function Enrichment Analyses of the Prognostic RNAs

Due to the prognostic RNAs selected from different modules, their associations may not be reflected by the WGCNA. Thus, we also used the cor.test function (https://stat.ethz.ch/R-manual/R-devel/library/stats/html/cor.test.html) in R to calculate the Pearson correlation coefficients (PCC) between prognostic lncRNAs and all module DERs and reconstructed the coexpression network using the Cytoscape software (version 3.6.1; http://www.cytoscape.org/).

The biological processes and pathways of genes in the coexpression network were predicted using the online Database for Annotation, Visualization, and Integrated Discovery (DAVID) (version 6.8; http://david.abcc.ncifcrf.gov) [25]. Significant Gene Ontology (GO) terms and Kyoto Encyclopedia of Genes and Genomes (KEGG) pathways were selected based on the threshold value of *p* value < 0.05.

## 3. Results

### 3.1. WGCNA Analysis Identifies PCa-Driven Modules

**Figure 1:**
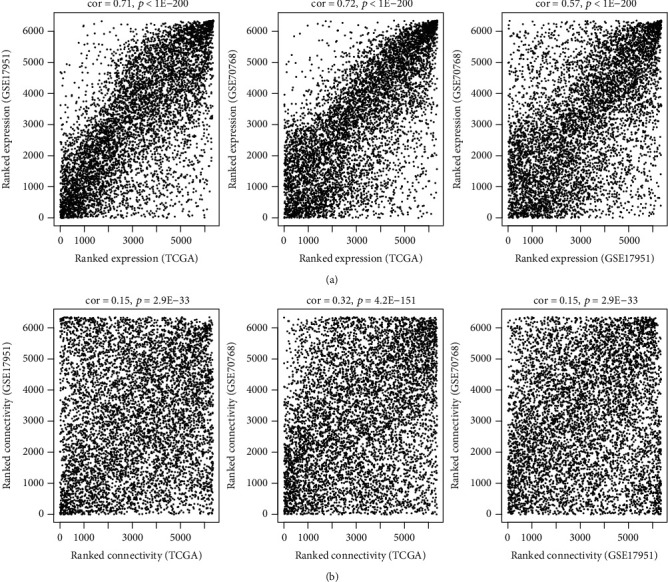
Significant correlations between any two datasets (TCGA, GSE17951, and GSE7076). (a) The RNA expression levels. (b) The connectivity.

**Figure 2:**
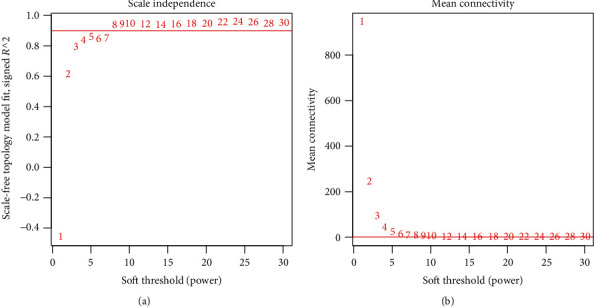
Soft threshold power *β*. (a) Soft threshold power *β* selected when the *R*^2^ reached 0.9 for the first time. (b) The mean connectivity corresponding to different *β* values.

**Figure 3:**
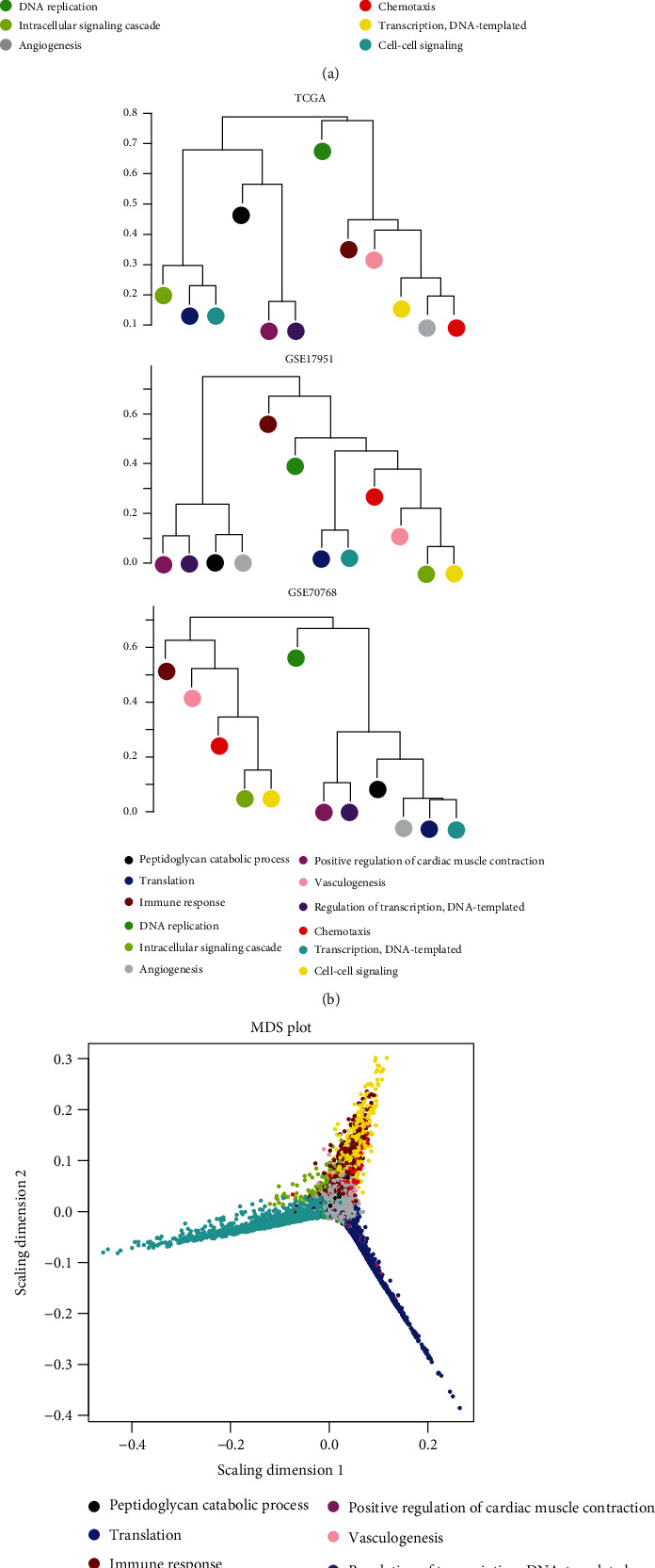
Coexpression modules screened based on WGCNA analysis. (a) Clustering dendrogram of gene coexpression modules from TCGA, GSE17951, and GSE7076 datasets. (b) A dendrogram of the module eigengenes from TCGA, GSE17951, and GSE7076 datasets. (c) A multidimensional scaling (MDS) plot of the module eigengene from TCGA datasets.

**Figure 4:**
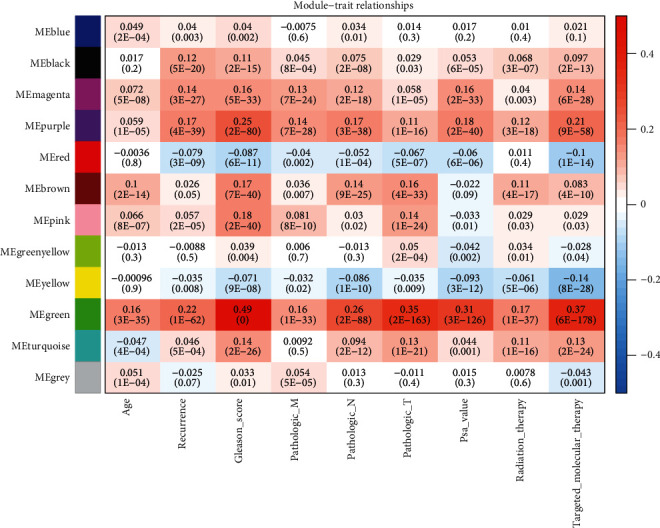
Heatmap of the correlation between module eigengenes and clinical traits.

After data processing and annotation, 695 lncRNAs and 6,613 protein-encoding mRNAs were identified to be shared within TCGA, GSE17951, and GSE7076 datasets. Thus, they were used for the WGCNA analysis. Correlation analysis showed that there were significantly positive correlations of RNAs between any two datasets irrespective of the expression level (Figure 1(a)) or the connectivity (Figure 1(b)), indicating these datasets were comparable. The soft threshold power *β* was selected as 8 according to the criterion of the scale-free topology (*R*^2^ = 0.9) (Figure 2(a)), using which the mean connectivity for the network was calculated (=1) (Figure 2(b)). A total of 12 modules were identified using TCGA dataset after the DynamicTreeCut analysis (Figures 3(a) and 3(b); Table 1), which was also validated in the GSE17951 and GSE7076 datasets (Figures 3(a) and 3(b)). The black module contained 99 genes (60 mRNAs and 39 lncRNAs); the blue module contained 964 genes (919 mRNAs and 45 lncRNAs); the brown module contained 422 genes (408 mRNAs and 14 lncRNAs); the green module contained 138 genes (134 mRNAs and 4 lncRNAs); the green-yellow module contained 71 genes (69 mRNAs and 2 lncRNAs); the grey module contained 1,705 genes (1,524 mRNAs and 181 lncRNAs); the magenta module contained 78 genes (67 mRNAs and 11 lncRNAs); the pink module contained 78 genes (74 mRNAs and 4 lncRNAs); the purple module contained 74 genes (50 mRNAs and 24 lncRNAs); the red module contained 129 genes (121 mRNAs and 8 lncRNAs); the turquoise module contained 1,491 genes (1,446 mRNAs and 45 lncRNAs); and the yellow module contained 412 genes (388 mRNAs and 24 lncRNAs) (Table 1). From Figure 3(c), we could see that the RNAs belonged to the similar module tended to group together (such as blue and turquoise). Among these 12 modules, blue, brown, green, pink, red, and yellow were considered to be preserved and may be PCa-driven (Table 1). This conclusion may be believable because there were also significant associations between these modules genes and clinical characteristics of PCa patients (Figure 4). the Blue module was significantly associated with Age, recurrence, Gleason_score, and Pathologic_N; the brown module was significantly associated with Age, Gleason_score, Pathologic_M, Pathologic_N, Pathologic_T, Radiation_therapy, and Targeted_molecular_therapy; the red module was significantly associated with recurrence, Gleason_score, Pathologic_M, Pathologic_N, Pathologic_T, prostate-specific antigen (PSA)_value, and Targeted_molecular_therapy; the yellow module was significantly associated with recurrence, Gleason_score, Pathologic_M, Pathologic_N, Pathologic_T, PSA_value, Radiation_therapy, and Targeted_molecular_therapy; and the green and pink modules were associated with all parameters (Figure 4).

### 3.2. The Venn Analysis Identifies Differentially Expressed PCa-Driven Module Genes

**Figure 5:**
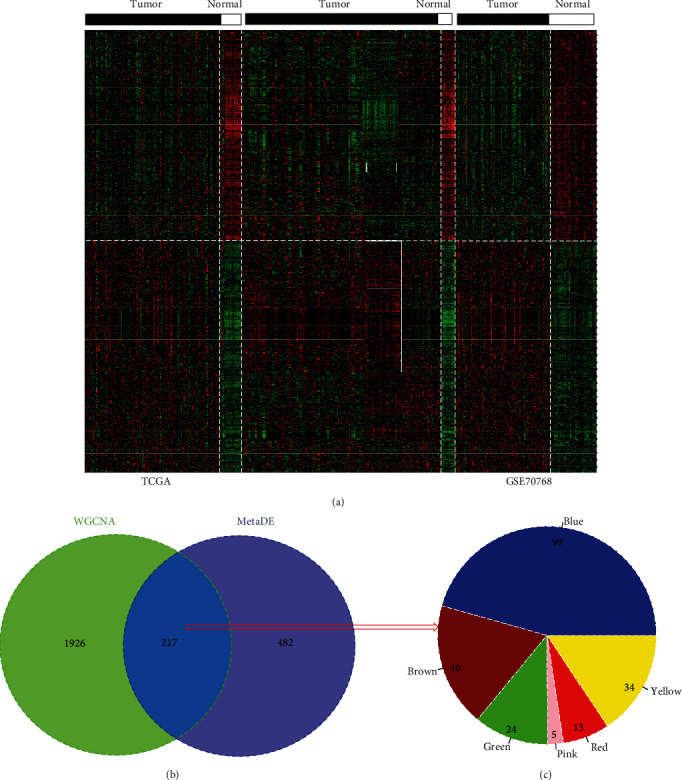
Identification of differentially expressed module genes. (a) Heatmap of differentially expressed RNAs in three datasets. (b) The Venn diagram to obtain the overlap between differentially expressed RNAs and module genes.

A total of 699 RNAs were identified as DERs in analysis of all three datasets (TCGA, GSE17951, and GSE7076), including 461 upregulated (37 DELs; 424 DEGs) and 238 downregulated (21 DELs; 217 DEGs).  Figure 5(a) shows the samples were distinctly separated according to the expression (high, red; low, green) of these DERs, and the differential pattern was similar among different datasets, indicating these genes may be representative for PCa. These 699 genes were subsequently overlapped with the 2,143 genes of 12 PCa-driven modules. The results showed 217 (including 12 lncRNAs and 205 mRNAs) were common, containing 99 of the blue module, 40 of the brown module, 24 of the green module, 5 of the pink module, 15 of the red module, and 34 of the yellow module (Figure 5(b)), suggesting these 217 genes may be key PCa-driven module genes and may represent alternative biomarkers.

### 3.3. The LASSO Analysis Identifies a 9-Gene Signature for the Prognosis Prediction

The univariate Cox regression analysis revealed that 33 module DERs (including 26 DEGs and 7 DELs) were significantly related to OS (log-rank *p* < 0.05). Then, these 33 DERs were entered into the multivariate Cox regression model. As a result, 9 DERs (including 6 DEGs and 3 DELs) were identified as the independent predictors of OS. A subsequent LASSO analysis confirmed these 9 DERs from blue, green, and yellow modules may constitute the optimal prognostic signature (DEL: DLG5-AS1 (DLG5 antisense RNA 1), MAGI2-AS3 (MAGI2 antisense RNA 3), and RHPN1-AS1 (RHPN1 antisense RNA 1); DEG: GINS2 (GINS complex subunit 2), NLGN2 (neuroligin 2), EBNA1BP2 (EBNA1 binding protein 2), MELK (maternal embryonic leucine zipper kinase, EIF5AL1 (eukaryotic translation initiation factor 5A like 1), and G6PC3 (glucose-6-phosphatase catalytic subunit 3)) (Table 2). According to the LASSO coefficients and the gene expression levels, the risk score was calculated for each patient as follows: risk score = (−0.2216 × expression of DLG5 − AS1) + (−0.7093 × expression of MAGI2 − AS3) + (−1.5300 × expression of RHPN1 − AS1) + (1.4561 × expression of GINS2) + (1.7956 × expression of NLGN2) + (2.7422 × expression of EBNA1BP2) + (−0.0482 × expression of MELK]) + (−1.5588 × expression of EIF5AL1) + (−1.5551 × expression of G6PC3).

**Figure 6:**
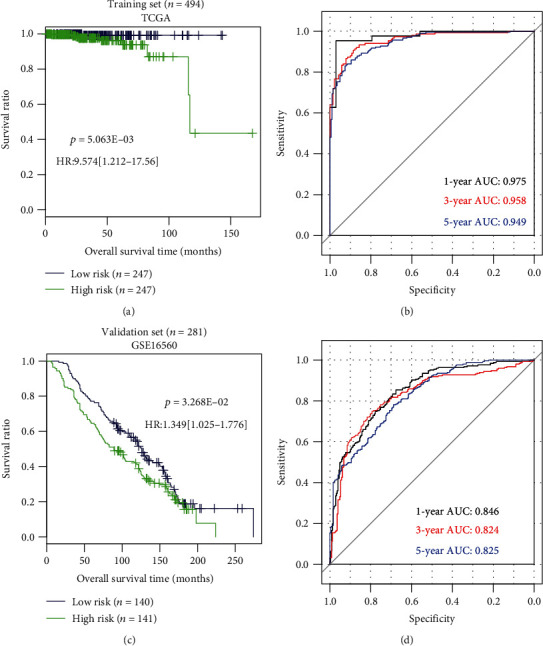
The prediction performance assessment of the prognostic signature. (a) The Kaplan–Meier survival curve analysis of TCGA dataset. (b) The receiver operator characteristic (ROC) curve analysis of TCGA dataset. (c) The Kaplan–Meier survival curve analysis of the GSE16560 dataset. (d) The receiver operator characteristic curve analysis of the GSE16560 dataset. HR: hazard ratio; AUC: area under the ROC curve.

**Figure 7:**
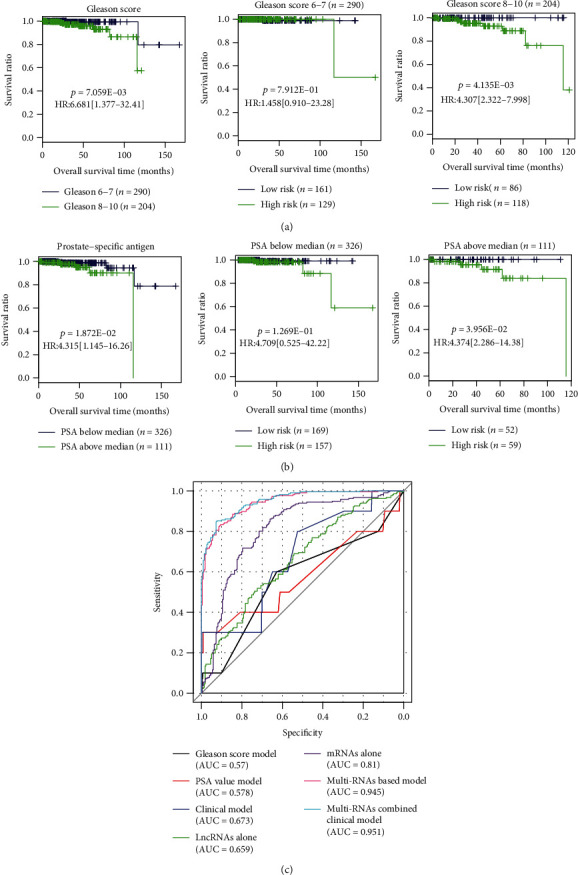
The superiority of the molecular prognostic signature to clinical indicators. (a) Stratification analysis for the Gleason score. (b) Stratification analysis for the level of prostate-specific antigen (PSA). (c) Time-dependent ROC curve analysis constructed according to various models. HR: hazard ratio; AUC: area under the receiver operator characteristic curve.

The patients were divided into the two groups by their corresponding risk scores (low-risk group, <median; high-risk group, ≥median). The Kaplan–Meier plots showed that patients having a higher risk score possessed a significantly worse OS compared with those having a lower risk score (TCGA: HR = 9.574, 95%CI = 1.212 − 17.56; *p* = 5.063E − 03; GSE16560: HR = 1.349, 95%CI = 1.025 − 1.776; *p* = 3.268E − 02) (Figure 6). ROC curves of the training dataset TCGA showed that the AUC at 1, 3, and 5 years was 0.975, 0.958, and 0.948, respectively; while for the validation dataset GSE16560, the AUC at 1, 3, and 5 years was 0.846, 0.824, and 0.825, respectively (Figure 6). These findings indicated this 9-gene-based risk score model can effectively separate the prognosis of patients with high accuracy. Moreover, univariate and multivariate Cox regression analyses demonstrated the prognostic value of the risk score was independent of the Gleason score and PSA (Table 3). Also, the prognosis of patients with the same Gleason score (8-10) (Figure 7(a)) and the level of PSA (above median) (Figure 7(b)) could be further separated by the risk score, implying the prognostic performance of molecular biomarker-based risk score was higher than that of the clinical model, which was also proved according to the time-dependent ROC curve analysis (Figure 7(c)).

### 3.4. The DAVID Analysis Identifies the Function of Prognostic Genes

**Figure 8:**
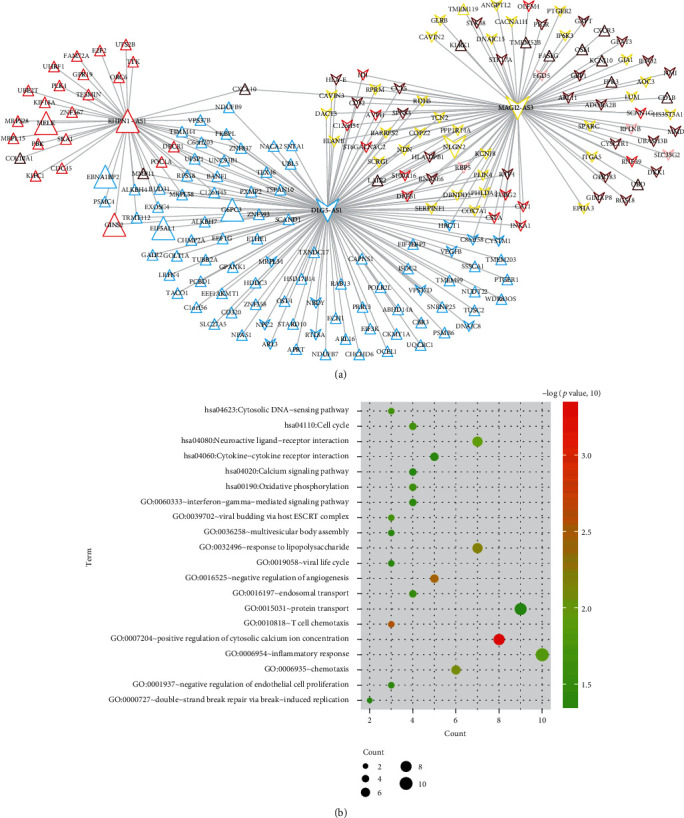
Function analysis for the prognostic genes. (a) A coexpression network between three prognostic lncRNAs and module differentially expressed mRNAs. Triangle, upregulated; inverted triangle, downregulated. The different colors corresponded to the module color. The larger nodes were the signature RNAs. (b) The DAVID enrichment analysis. GO: Gene Ontology.

After calculation of the PCC between three prognostic DELs and module DEGs, a total of 259 relationship pairs were considered to be correlated due to their absolute value of PCC > 0.4. These relationship pairs were used to construct the coexpression network (Figure 8(a)), from which we could see that downregulated MAGI2-AS3 may coexpress with SPARC (secreted protein acidic and cysteine rich) and GJA1 (gap junction protein alpha 1) of the yellow module and CYSLTR1 (cysteinyl leukotriene receptor 1) of the brown module; downregulated DLG5-AS1 may coexpress with VPS37D (VPS37D subunit of ESCRT-I), EIF5AL1, and G6PC3 of the blue module or DEFB1 (defensin beta 1) of the red module; RHPN1-AS1 may coexpress with MELK, GINS2, ORC6 (origin recognition complex subunit 6), and CDC45 (cell division cycle 45) of the green module and EBNA1BP2 of the blue module. The DAVID enrichment analysis identified 14 GO biological process terms, including GO:0007204~positive regulation of cytosolic calcium ion concentration (GJA1), GO:0016525~negative regulation of angiogenesis (SPARC), GO:0032496~response to lipopolysaccharide (SPARC), GO:0006935~chemotaxis (DEFB1), GO:0039702~viral budding via host ESCRT complex (VPS37D), GO:0001937~negative regulation of endothelial cell proliferation (SPARC), GO:0019058~viral life cycle (VPS37D), and GO:0000727~double-strand break repair via break-induced replication (GINS2) (Table 4; Figure 8(b)). In addition, 6 KEGG pathways were also enriched, such as hsa04080:Neuroactive ligand-receptor interaction (CYSLTR1), hsa04110:Cell cycle (CDC45, ORC6), and hsa04020:Calcium signaling pathway (CYSLTR1) (Table 4; Figure 8(b)).

## 4. Discussion

Using the training and validation datasets, we first identified 6 preserved PCa-driven modules and then screened 9 prognosis-related genes (including 3 lncRNAs: DLG5-AS1, MAGI2-AS3, and RHPN1-AS1; and 6 mRNAs: GINS2, NLGN2, EBNA1BP2, MELK, EIF5AL1, and G6PC3) from these modules to construct the risk score. The ROC curve analysis demonstrated the prediction accuracy of this molecular risk score was higher than that of clinical indicators (the Gleason score [AUC = 0.945 vs.0.57], PSA [AUC = 0.945 vs.0.578], and combined [AUC = 0.945 vs.0.673]), which was in line with the studies of Li et al. [9], Shi et al. [10], Huang et al. [11], and Xu et al. [12]. More importantly, our integrated model seemed to be more effective than the single mRNA model (Xu et al.: 4-mRNA, AUC = 0.945 vs.0.904 [26]) for OS prediction, which was also observed in our study (AUC = 0.945 vs.0.81) (Figure 7). Although there was no study to investigate the prognostic ability of the lncRNA signature for OS, their lower prognostic performance for BCR-free survival (Huang et al.: AUC = 0.823 [11]; Xu et al.: AUC = 0.79 [12]) may indirectly confirm the prognostic significance of the combined signature compared with the single lncRNA type, which was also reflected by our study (AUC = 0.945 vs.0.659) (Figure 7).

Among these 9 genes, four of them had the consistency between the expression level and the expected prognosis results, that is, the high expression of oncogenes (GINS2 and EBNA1BP2: upregulated, HR > 1) predicted the worse OS, while the high expression of tumor suppressor genes (MAGI2-AS3 and DLG5-AS1: downregulated, HR < 1) predicted the better OS. These findings indicated these four genes may be especially crucial therapeutic targets for PCa. Although rare studies identified lncRNA MAGI2-AS3 as a prognostic biomarker for PCa, the reports of other cancers can indirectly explain their roles. For example, Liu et al. observed that MAGI2-AS3 was downregulated in breast cancer tissues compared to normal adjacent tissues [27]. Overexpression of MAGI2-AS3 suppressed the proliferative, migratory, and invasive capability, while promoted the apoptosis of lung squamous cell carcinoma [28], bladder cancer [29], breast cancer [27, 30], and hepatocellular carcinoma cells [31]. Downregulated MAGI2-AS3 was significantly associated with tumor size, lymph node metastasis, TNM stage, and poor OS [29, 31, 32]. Most of the studies revealed MAGI2-AS3 may function in cancers as a competing endogenous RNA for miRNAs (such as miR-374a/b-5p [28, 31], miRNA-23a-3p [33], and miR-15b-5p [29]) to regulate their target genes (such as CADM2 [28], SMG1 [31], PTEN [33], and CCDC19 [29]), while few indicated MAGI2-AS3 may directly interact with target gene KDM1A [34]. However, the mechanism of MAGI2-AS3 remained unclear. In this study, we predicted that downregulated MAGI2-AS3 may be involved in PCa by leading to the low expression of inflammation (SPARC) or calcium signaling pathway related genes (GJA1 and CYSLTR1). This hypothesis may be believable because these target genes have been implicated to be associated with PCa or other cancers. SPARC expression was found to be decreased in PCa cell lines, the mechanism of which may be attributed to the hypermethylation of its promoter. Also, hypermethylation level was shown to be correlated with a poor prognosis [35]. PCa cells treated with exogenous SPARC exhibited significantly decreased proliferative and migratory abilities [36]. GJA1 (also known as connexin 43) expression was measured to be significantly reduced in tumor tissues compared to that of benign prostatic hyperplasia. Reduced GJA1 expression was associated with high levels of preoperative PSA, high Gleason score, and advanced pT stage and was an independent predictor for shortened postoperative BCR-free survival [37]. Forced expression of GJA1 induced apoptosis of PCa cells by downregulation of Bcl-2 expression and upregulation of caspase-3 activity [38]. By immunohistochemistry (IHC) and quantitative reverse transcription-polymerase chain reaction (qRT-PCR) analyses, Venerito et al. found CYSLTR1 expression was decreased by 0.26-fold in esophageal squamous cell cancer tissues compared to control mucosa [39]. Also, the roles of GINS2 and EBNA1BP2 in PCa could be reflected by their associations with other cancers. GINS2 was shown to be upregulated in the cervical cancer cell lines and tumor specimens compared to the normal control. Patients with higher GINS2 expression had shorter OS than those with lower GINS2 expression [40]. Downregulation of GINS2 markedly inhibited cell proliferation, migration, and invasion [40] and increased the apoptosis rate [41]. Total saponins from *Paris forrestii* (Takht) H. Li. exhibited an anticancer effect on PCa cells by downregulating GINS2 [42]. Both protein and mRNA levels of EBNA1BP2 were reported to be upregulated in lung cancer samples. EBNA1BP2 may promote cancer cell proliferation by blocking the degradation of oncogene c-Myc [43]. lncRNA DLG5-AS1 may be a newly identified biomarker and therapeutic target for cancer because there was no evidence to show its association with cancer. In this study, we predicted downregulated DLG5-AS1 may exert roles in PCa by decreasing the transcription of DEFB1. This theory may be credible because DEFB1 had been found to be significantly downregulated in PCa tissues and three cell lines [44, 45]. The low expression of DEFB1 in PCa may be mediated by the hypermethylation of the 14 CpG dinucleotide units in its 5′-end promoter region [44]. High expression of DEFB1 was reported to correlate with better prognosis in patients with renal cell carcinoma [46].

The inconsistency between the expression level and the expected prognosis in the other five genes (RHPN1-AS1, MELK, EIF5AL1, and G6PC3: upregulated, but HR < 1; NLGN2: downregulated, but HR > 1) may be attributed to a protective response mechanism in order to resist the development of cancer. This speculation can be verified based on the function studies of these genes. Knockdown of RHPN1-AS1 was shown to result in the development of gefitinib resistance in non-small cell lung cancer cells, whereas the overexpression of RHPN1-AS1 sensitized gefitinib resistant NSCLC cells to gefitinib treatment. Decreased expression of RHPN1-AS1 was associated with poor prognosis of non-small cell lung cancer patients [47]. Furthermore, we also predicted RHPN1-AS1 can positively coexpress with CDC45 and ORC. Patients with low expression of CDC45 and ORC6 were also demonstrated to have significantly worse relapse-free survival and OS [48]. Mass spectrometry identified G6PC3 was a downstream target of Coronin 3. High expressed Coronin 3 was reported to promote the progression of hepatocellular carcinoma cells by inhibiting the expression of G6PC3 [50]. The roles of other genes needed further investigation in the future.

Some limitations of our study should be acknowledged. First, this is a study to validate the prognostic value of our identified molecular signature using the retrospective data from the public-available dataset. Prospective clinical trials should be designed to further confirm its prediction ability. Second, wet experiments (IHC, qRT-PCR, overexpression, or knockdown) should be performed to elucidate the expression and roles of the signature genes in PCa because most of them were not reported previously and some were even contradictory. Third, the coexpression relationship between lncRNAs and mRNAs should be explored by chromatin immunoprecipitation, RNA immunoprecipitation, and biotin-labeled RNA pull-down assays [49].

## 5. Conclusion

Using the WGCNA and LASSO methods, we developed a nine-RNA (including 3 lncRNAs and 6 mRNAs) prognostic signature for PCa patients. This risk score could independently predict the OS and further discriminate the prognostic outcomes for patients with the Gleason score (8-10) and the high level of PSA (above median). Besides, our study may provide new therapeutic targets for PCa patients and the underlying mechanisms for them (MAGI2-AS3-SPARC/GJA1/CYSLTR1, DLG5-AS1-DEFB1, and RHPN1-AS1-CDC45/ORC).

## Figures and Tables

**Table 1:** Preserved modules identified based on weighted gene coexpression network analysis.

ID	Color	Module size	mRNA	lncRNA	Preservation *Z*-score	Module annotation
Module 1	Black	99	60	39	0.2408	Peptidoglycan catabolic process
Module 2	Blue	964	919	45	**8.9080**	Translation
Module 3	Brown	422	408	14	**19.2639**	Immune response
Module 4	Green	138	134	4	**11.3671**	DNA replication
Module 5	Green-yellow	71	69	2	4.0398	Intracellular signaling cascade
Module 6	Grey	1705	1524	181	1.0886	Angiogenesis
Module 7	Magenta	78	67	11	4.6283	Positive regulation of cardiac muscle contraction
Module 8	Pink	78	74	4	**9.9431**	Vasculogenesis
Module 9	Purple	74	50	24	4.3446	Regulation of transcription, DNA-templated
Module 10	Red	129	121	8	**8.9918**	Chemotaxis
Module 11	Turquoise	1491	1446	45	1.9359	Transcription, DNA-templated
Module 12	Yellow	412	388	24	**38.0674**	Cell-cell signaling

Bold indicated preserved modules with preservation *Z* − score > 5.

**Table 2:** The optimal prognostic signature.

Symbol	Module	Expression	Type	Univariate Cox regression			Multivariate Cox regression			LASSO coefficient
				HR	95% CI	*p* value	HR	95% CI	*p* value	
GINS2	Green	Upregulated	mRNA	3.248	1.344-7.849	4.450E-03	1.399	1.261-2.036	3.33E-02	1.4561
NLGN2	Yellow	Downregulated	mRNA	6.514	1.237-14.31	1.350E-02	3.012	2.000-9.079	2.10E-02	1.7956
EBNA1BP2	Blue	Upregulated	mRNA	7.112	1.048-18.28	2.250E-02	4.470	2.904-12.115	2.79E-02	2.7422
DLG5-AS1	Blue	Downregulated	lncRNA	0.629	0.199-0.987	3.655E-02	0.589	0.290-0.931	4.59E-02	-0.2216
MAGI2-AS3	Yellow	Downregulated	lncRNA	0.354	0.0572-0.914	2.210E-02	0.512	0.220-0.913	4.51E-02	-0.7093
RHPN1-AS1	Green	Upregulated	lncRNA	0.272	0.0286-0.581	2.210E-02	0.513	0.236-0.711	3.89E-02	-1.5300
MELK	Green	Upregulated	mRNA	0.557	0.047-0.625	1.950E-02	0.360	0.110-1.179	3.84E-02	-0.0482
EIF5AL1	Blue	Upregulated	mRNA	0.194	0.0311-0.414	4.000E-02	0.540	0.273-0.701	3.26E-02	-1.5588
G6PC3	Blue	Upregulated	mRNA	0.237	0.0617-0.912	1.800E-02	0.352	0.1117-0.509	2.66E-02	-1.5551

HR: hazard ratio; CI: confidence interval; LASSO: least absolute shrinkage and selection operator.

**Table 3:** The univariate and multivariate Cox regression analysis to identify independent prognostic factors.

Variables	TCGA (*n* = 494)	Univariate analysis			Multivariate analysis		
		HR	95% CI	*p* value	HR	95% CI	*p* value
Age (years, mean ± SD)	61.03 ± 6.84	1.053	0.956-1.160	2.91E-01	—	—	—
Pathologic_M (M0/M1/-)	452/3/39	2.892	0.470-5.366	1.48E-01	—	—	—
Pathologic_N (N0/N1/-)	344/78/72	3.609	0.799-16.30	7.46E-02	—	—	—
Pathologic_T (T2/T3/T4/-)	186/291/10/7	2.242	0.601-8.373	2.27E-01	—	—	—
Radiation therapy (yes/no/-)	59/386/49	2.984	0.577-15.42	2.33E-01	—	—	—
Targeted molecular therapy (yes/no/-)	52/392/50	3.127	0.592-16.52	2.20E-01	—	—	—
Gleason score (6/7/8/9/10)	45/245/63/137/4	2.959	1.337-6.549	**2.28E-03**	1.685	1.163-3.963	**2.32E-02**
Prostate-specific antigen	1.75 ± 15.89	1.062	1.004-1.124	**9.60E-05**	1.022	1.019-1.054	**1.52E-02**
Recurrence (yes/no/-)	368/58/68	7.224	1.937-26.94	**5.68E-04**	2.081	0.424-10.222	3.67E-01
Prognostic score status (high/low)	247/247	9.574	1.212-17.56	**5.06E-03**	5.846	1.708-18.27	**1.01E-02**
Death (dead/alive)	10/484	—	—	—	—	—	—
Overall survival days (months, mean ± SD)	36.10 ± 26.32	—	—	—	—	—	—

SD: standard deviation; TCGA: The Cancer Genome Atlas; HR: hazard ratio; CI: confidence interval. Bold indicated the factors with statistical significance.

**Table 4:** Function enrichment analysis.

Category	Term	Count	*p* value	Genes
GO_BP	GO:0007204~positive regulation of cytosolic calcium ion concentration	8	4.15E-04	PTGER1, PTGER2, CYSLTR1, GALR2, GJA1, CD52, FPR3, and CXCR3
GO_BP	GO:0010818~T cell chemotaxis	3	2.71E-03	GPR183, CXCR3, and CXCL10
GO_BP	GO:0016525~negative regulation of angiogenesis	5	3.51E-03	SERPINF1, FASLG, CXCR3, SPARC, and CXCL10
GO_BP	GO:0032496~response to lipopolysaccharide	7	6.37E-03	PTGER1, PTGER2, KCNJ8, ELANE, FASLG, SPARC, and CXCL10
GO_BP	GO:0006935~chemotaxis	6	7.84E-03	RARRES2, CYSLTR1, CXCR3, CCL5, DEFB1, and CXCL10
GO_BP	GO:0006954~inflammatory response	10	1.47E-02	TUSC2, PTGER1, PTGER2, RARRES2, NMI, FPR3, CXCR3, CCL5, CXCL10, and AOC3
GO_BP	GO:0039702~viral budding via host ESCRT complex	3	2.04E-02	CHMP2A, VPS37B, and VPS37D
GO_BP	GO:0016197~endosomal transport	4	2.89E-02	CHMP2A, VPS37B, VPS37D, and RAB13
GO_BP	GO:0001937~negative regulation of endothelial cell proliferation	3	3.42E-02	GJA1, CXCR3, and SPARC
GO_BP	GO:0060333~interferon-gamma-mediated signaling pathway	4	3.48E-02	NMI, HLA-DPB1, HLA-E, and GBP1
GO_BP	GO:0019058~viral life cycle	3	3.87E-02	CHMP2A, VPS37B, and VPS37D
GO_BP	GO:0036258~multivesicular body assembly	3	3.87E-02	CHMP2A, VPS37B, and VPS37D
GO_BP	GO:0015031~protein transport	9	4.67E-02	CHMP2A, COPZ2, DNAJC15, GOLT1A, EIF5AL1, KIF18A, VPS37B, VPS37D, and NACA2
GO_BP	GO:0000727~double-strand break repair via break-induced replication	2	4.93E-02	GINS2, CDC45
KEGG	hsa04080:Neuroactive ligand-receptor interaction	7	1.10E-02	PTGER1, PTGER2, GLRB, CYSLTR1, ADORA2B, GALR2, and FPR3
KEGG	hsa04110:Cell cycle	4	2.16E-02	E2F2, CDC45, TTK, and ORC6
KEGG	hsa04623:Cytosolic DNA-sensing pathway	3	2.21E-02	POLR2L, CCL5, and CXCL10
KEGG	hsa00190:Oxidative phosphorylation	2.51	1.10E-02	UQCRC1, COX7A1, NDUFB7, and NDUFB9
KEGG	hsa04060:Cytokine-cytokine receptor interaction	5	3.86E-02	OSM, FASLG, CXCR3, CCL5, and CXCL10
KEGG	hsa04020:Calcium signaling pathway	4	4.43E-02	PTGER1, CYSLTR1, ADORA2B, and CACNA1H

GO: Gene Ontology; BP: biological process; KEGG: Kyoto Encyclopedia of Genes and Genomes.

## Data Availability

All data were downloaded from The Cancer Genome Atlas (TCGA, https://portal.gdc.cancer.gov/) and Gene Expression Omnibus (GEO, http://www.ncbi.nlm.nih.gov/geo/).
